# Non-genetic Heterogeneity of Macrophages in Diseases—A Medical Perspective

**DOI:** 10.3389/fcell.2020.613116

**Published:** 2020-12-14

**Authors:** Grégoire Gessain, Camille Blériot, Florent Ginhoux

**Affiliations:** ^1^Université de Paris, Faculté de Santé, Paris; ^2^Inserm U1015, Gustave Roussy, Villejuif, France; ^3^Singapore Immunology Network(SIgN), Agency for Science, Technology and Research (A^∗^STAR), Biopolis, Singapore, Singapore; ^4^Shanghai Institute of Immunology, Shanghai Jiao Tong University School of Medicine, Shanghai, China; ^5^Translational Immunology Institute, SingHealth Duke-NUS Academic Medical Centre, Singapore, Singapore

**Keywords:** macrophage, monocyte, heterogeneity, human diseases, ontogeny, tissue-resident, inflammation

## Abstract

Macrophages are sessile immune cells with a high functional plasticity. Initially considered as a uniform population of phagocytic scavengers, it is now widely accepted that these cells also assume developmental and metabolic functions specific of their tissue of residence. Hence, the paradigm is shifting while our comprehension of macrophage heterogeneity improves. Accordingly, exploiting this intrinsic versatility appears more and more promising for the establishment of innovative therapeutic strategies. Nevertheless, identifying relevant therapeutic targets remains a considerable challenge. Herein, we discuss various features of macrophage heterogeneity in five main categories of human diseases: infectious, inflammatory, metabolic, age-related, and neoplastic disorders. We summarize the current understanding of how macrophage heterogeneity may impact the pathogenesis of these diseases and propose a comprehensive overview with the aim to help in establishing future macrophage-targeted therapies.

## Introduction

Macrophages are sessile within tissues at steady state and are therefore often named resident tissue macrophages (RTMs). They are considered as the guardians of tissue integrity due to their ability to phagocyte any “non-self” intruders and damaged or dying “self” cells. However, narrowing macrophages to their role of tissue scavengers appears too reductive as more and more immune and non-immune functions are documented (Okabe and Medzhitov, 2016). For example, a very recent study has reported that cardiac macrophages were involved in the elimination of dysfunctional mitochondria ejected from cardiomyocytes (Nicolás-Ávila et al., 2020), a mandatory task to the maintenance of heart homeostasis. It illustrates how macrophage-specific phagocytic abilities have been selected and shaped during evolution. Thus, RTMs should now be more considered as fully integrated and tissue-supportive components of any given tissue rather than only protective innate immune cells. Furthermore, even focusing on their immune functions, phagocytosis of foreign bodies appears only as a single string on their functional bow. Indeed, RTMs (and dendritic cells) have been anticipated as positive initiators of immunity (Janeway, 1989), assuming the original recognition of non-self-antigens. This process was proposed to lead to the generation of second signals strictly required for an efficient adaptive immune response, and these brilliant hypotheses have been convincingly demonstrated since (Fearon and Locksley, 1996; Hoffmann et al., 1999). So, without RTMs, efficiency of the response as well as immune memory would be altered.

Twenty years ago, the biology of RTMs has been dichotomized into the so-called pro-inflammatory M1 and anti-inflammatory M2 states (Mills et al., 2000). Although outdated since, this was the first conceptual step toward the recognition of the complexity of macrophage biology (Bleriot et al., 2020). Nowadays, it has been clearly demonstrated that RTMs were not only bipolar but could actually harbor a full spectrum of activation states as an integrative response to any signals received (Xue et al., 2014; Ginhoux et al., 2016; Glass and Natoli, 2016). Therefore, RTMs display a plasticity that could be at least comparable or even more pronounced than the one well-recognized for other immune cells such as lymphocytes. It appears fundamental to uncover how this remarkable heterogeneity is generated and how it is modulated in several pathological conditions. Many recent studies described uncharacterized subpopulations of RTMs involved in several pathologies (Chakarov et al., 2019; Jaitin et al., 2019; Ramachandran et al., 2019; Xiong et al., 2019; Zilionis et al., 2019; Katzenelenbogen et al., 2020; Molgora et al., 2020). However, as the other immune and non-immune cells from a same organism, it is essential to remind that macrophages are genetically identical. Thus, to understand this diversity of phenotypes and functions, we must identify factors shaping macrophage identities and responses to stimulation. We have recently proposed to break down these parameters into four interconnected cardinal points, namely, origin, location, time of residence and tissue inflammatory status (Bleriot et al., 2020).

**FIGURE 1:**
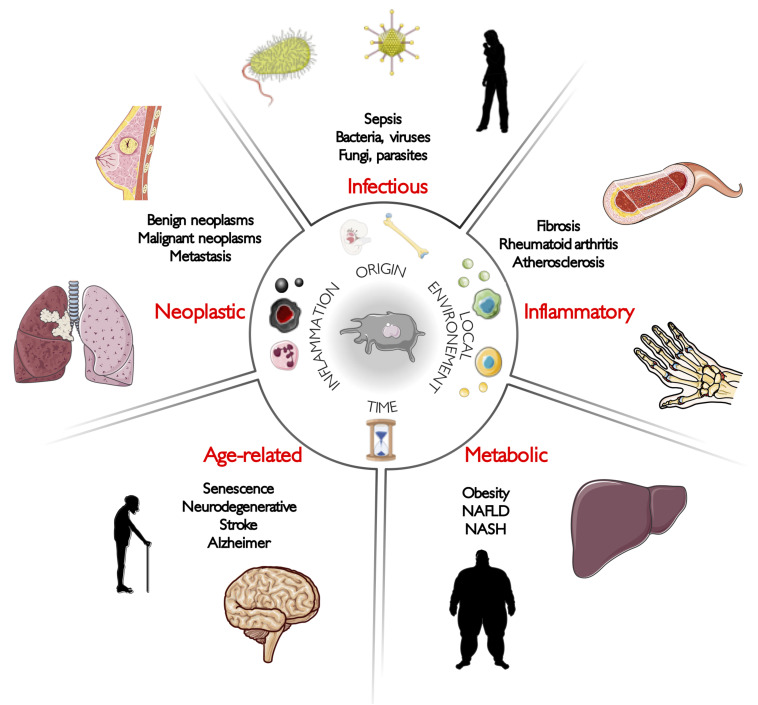
Macrophages are at the crossroads of diseases. Macrophages are implicated in the pathogenesis of almost all human diseases. Here, our aim is to illustrate their non-genetic heterogeneity by describing recent macrophage-related discoveries in five main categories of human diseases: infectious, inflammatory, metabolic, age-related, and neoplastic disorders.

Among these parameters, it is necessary to recall that macrophages are the first immune cells to seed tissues during embryogenesis, as most of them do not derive from adult blood monocytes as it was commonly assumed for decades, but actually derive from embryonic precursors (Ginhoux et al., 2010; Schulz et al., 2012; Hashimoto et al., 2013; Yona et al., 2013; Gomez Perdiguero et al., 2015). Therefore, they are the immune cells forging the earliest ties with their tissue of residence. Indeed, it has been shown that even originating from common embryonic ancestors that could be designed as pre-macrophages, RTMs acquire very early tissue-specific programs depending of their local environment during fetal development (Mass et al., 2016). However, it has been also demonstrated that circulating monocytes seeding adult tissue to give rise to adult RTM also undergo a dramatic reprogramming reflecting the integration of tissue specificities (Bonnardel et al., 2019; Sakai et al., 2019). These diverse environmental cues that drive macrophage differentiation are unique to the niche of residence and involved a tissue-specific cocktail of different cytokines, metabolites, chemokines, and direct cell interactions. Although attempts have been made to describe these environmental programs (T’Jonck et al., 2018), they remain far from being fully described in an extensive manner. This being said, the central point becomes to decipher programs driving macrophage biology and how they evolve across time in healthy tissues or during disease development. In this review, we have split diseases into five main categories: infectious, inflammatory, metabolic, age-related, and neoplastic disorders (Figure 1). We discussed thereafter how macrophage biology is profoundly altered when homeostasis is disrupted, and how these changes can support pathogenesis.

## Macrophage Heterogeneity in Infectious Disorders

### Macrophages and Sepsis

Sepsis is the result of an aberrant host response to infection leading to organ dysfunctions (Singer et al., 2016). Across the globe, more than 30 million cases of sepsis are reported per year and it is the principal cause of death in intensive care units (ICU) (Fleischmann et al., 2016; Reinhart et al., 2017). Of note, it is also a very expensive condition for hospitals, as its annual costs exceed 20 billion dollars in the United States only (Singer et al., 2016). For decades, sepsis mortality was supposed to result solely from an hyperactive inflammatory response leading to harmful effects for the host such as fever, hypotension, tachypnea, tachycardia, coagulation disorders, and multiple-organ failure (Singer et al., 2016). For this reason, preventing excessive inflammation was the main objective in order to find a treatment for sepsis patients. However, a paradigm shift has started to emerge during the last decade because it appeared that the main immune dysfunction associated with high mortality was in reality not an excessive immune activation, but rather a strong immunosuppression, termed “sepsis-induced immuno-paralysis” (Leentjens et al., 2013; Wang et al., 2014). From a clinician perspective, this new paradigm was surprising at first, but rapidly supported by convincing evidences. Indeed, because of sepsis-induced immuno-paralysis, patients are unable to recover from their primary infection and are more likely to develop secondary infections from opportunistic pathogens later on (Otto et al., 2011). As a result, many patients with sepsis do not die from the initial pro-inflammatory hit, but later, from a secondary or opportunistic infection associated with an immunosuppressed state (Muenzer et al., 2010; Boomer et al., 2012; Walton et al., 2014). Therefore, researchers have progressively focused on ways to overcome the immuno-paralysis by developing immuno-stimulatory drugs (Leentjens et al., 2013; Delano and Ward, 2016; Patil et al., 2016).

Resident tissue macrophages play a fundamental role in such immuno-paralysis (Roquilly et al., 2020). For instance, some of the earliest evidence that macrophages may be involved in immuno-paralysis came from experiment of endotoxin-induced tolerance (Foster et al., 2007). In this study, authors showed that long-term endotoxin exposure of macrophages resulted in tolerogenic immune response. Such tolerance was not a result of DNA mutations but was induced by epigenetic modifications. Indeed, recognition of endotoxin by its specific receptor triggers chromatin modifications such as histone acetylation or methylation resulting in silencing of genes coding for pro-inflammatory molecules, but priming of genes coding for antimicrobial peptides (Foster et al., 2007). Of note, the initial dose of the priming agent, i.e., endotoxin in the seminal study discussed here, and pathogen inoculum in primary infection for sepsis patients, appear to be critical: if a high dose of endotoxin triggered immuno-paralysis of macrophages, a continuous exposure to very low doses actually results in enhanced responses to subsequent challenges, a process called trained immunity (Netea et al., 2016; Lajqi et al., 2019).

Similarly, in a double-infection murine model, Roquilly and colleagues showed that after recovering from a primary pneumonia, alveolar macrophages (AMs) displayed poor phagocytic capacity for several weeks (Roquilly et al., 2020). These weakened AMs originated from embryonic resident AMs that experienced a tolerogenic reprogramming of epigenetic nature. Of interest, signal-regulatory protein α (SIRPα), a membrane glycoprotein expressed mainly by myeloid cells, was a major player in the induction of this tolerogenic training. Finally, it was confirmed that AMs from patients with systemic inflammation still harbored reprogramming alterations up to six months after the resolution of inflammation. Very interestingly, *in vitro* inhibition of SIRPα enhanced phagocytosis in monocytes extracted from patients, suggesting that such treatment could modulate epigenetic reprogramming of monocytes and macrophages. Huge efforts are still needed to fully understand the phenomenon of immune tolerance and trained immunity as ones could hypothesize such effects being pathogen-specific. Further studies will integrate this recent concept for a more efficient targeting of immuno-paralyzed cells and hopefully the design of more efficient clinical treatments.

### Macrophages and Bacterial Infection

Tuberculosis (TB) is an airborne infection that principally affects the lungs, caused by the bacterial pathogen *Mycobacterium tuberculosis*. In 2010, it remained a serious global health threat responsible for 1.2 million deaths (Lozano et al., 2012). In the lungs, two macrophage subsets have been described, the alveolar (AMs) and the interstitial macrophages (IMs), AMs being fetal liver-derived whereas IMs being mostly adult monocyte-derived (Chakarov et al., 2019). A recent study has focused on the respective contribution of AMs and IMs in TB pathogenesis (Huang et al., 2018). The authors showed that AMs and IMs mounted divergent responses upon *Mycobacterium tuberculosis* infection: AMs promoted bacterial growth whereas IMs restricted it. Thus, specific targeting of the embryonic-derived AMs could be an interesting avenue for therapeutic research.

Another clinically relevant bacterium that has contributed to elucidate macrophage heterogeneity is *Listeria monocytogenes* (*Lm*). *Lm* is a foodborne pathogen that causes human listeriosis, a systemic infection with one of the highest mortality rates (Charlier et al., 2017). After ingestion of contaminated food, bacteria survive and multiply in the intestinal lumen and actively cross the intestinal barrier. Then, it disseminates within the host (Nikitas et al., 2011) and crosses both the blood–brain barrier and the placental barrier, leading, respectively, to meningitis and encephalitis, as well as abortion and neonatal infection in pregnant women (Lecuit, 2020). *Lm* is a powerful model pathogen that has led to significant discoveries, such as macrophage activation (Mackaness, 1969; Pamer, 2004). Furthermore, in a model of liver infection, *Lm* was able to induce necroptosis of Kupffer cells (KCs), the liver embryonic RTMs (Blériot et al., 2015). Necroptosis of KCs was responsible for (i) the recruitment of bone-marrow pro-inflammatory monocytes to clear the infection and repopulate the empty KC niche and (ii) their conversion after bacterial clearance into liver RTMs that harbor anti-inflammatory phenotype and tissue repair functions. Such a model challenges the M1–M2 paradigm by showing that a single macrophage is actually highly plastic and can be reprogrammed from a M1-like to M2-like phenotype across the course of an infection according to changes in its microenvironment.

### Macrophages and Viral Infection

The recently identified severe acute respiratory syndrome coronavirus 2 (SARS-Cov-2) is an enveloped RNA betacoronavirus responsible for the still active coronavirus disease 2019 outbreak (Covid-19). Patients with severe Covid-19 have various clinical symptoms such as pneumonia with fever, cough, and dyspnea (Guan et al., 2020). As in many benign viral infections, the majority of patients presenting with mild disease mounts an efficient immune response (Thevarajan et al., 2020). However, severe viral pneumonia can lead to acute respiratory distress syndrome (ARDS), a complication that occurs in 15.6% of patients with severe Covid-19 (Guan et al., 2020). A disproportionate inflammatory response to the virus contributes to the severity of the symptoms and can result to death (Mehta et al., 2020). Bronchoalveolar fluid (BALF) analysis of patients suffering from severe Covid-19 showed an elevated proportion of mononuclear phagocytes accounting for 80% of total cells, as compared to 40% in healthy controls (Liao et al., 2020). Among them, a near-complete depletion of AMs and an enrichment of inflammatory monocyte-derived macrophages was observed. Such a dramatic decrease of AMs has been confirmed recently by analysis of BALF samples from patients with mild or severe Covid-19 (Silvin et al., 2020). Here again, the contribution to lung damage of embryonic-resident macrophages as opposed to monocyte-derived macrophages remains to be investigated. Interestingly, patients with severe Covid-19 had an accumulation of immuno-suppressive HLA-DR^low^ classical monocytes, a feature commonly found in other severe illnesses (Lukaszewicz et al., 2009), as well as a Covid-19-specific decrease of non-classical CD14^low^CD16^high^ monocytes. This decrease appears to be a very characteristic biological signature of patients with severe Covid-19, easily measurable with standard diagnostic flow cytometry available in hospitals. Therefore, such monocyte heterogeneity constitutes a valuable predictive biomarker in blood samples that could help to the early detection of severe Covid-19 patients (Schulte-Schrepping et al., 2020; Silvin et al., 2020).

In addition, macrophage diversity could also be involved in the relatively efficient response of children, as compared to adults, to Covid-19 (Castagnoli et al., 2020). Indeed, because the children’s immune system is relatively immature, they follow an intense vaccination program that involves several adjuvants. As recently discussed by Mantovani and Netea, adjuvants have been shown to induce trained immunity and thus boost antimicrobial function in myeloid cells (Castagnoli et al., 2020; Mantovani and Netea, 2020). Adjuvants are known to elicit an innate immune response in myeloid cells, which is also efficient against heterologous pathogens. As a consequence, innate immune cells have an increased non-specific response that goes well beyond the antigen in the vaccine (Netea et al., 2020a). Although still theoretical, this vaccination-induced reprogramming of myeloid cells could be one among many reasons why children are relatively resistant to Covid-19. Although others’ hypothesis are currently under study (Steinman et al., 2020), the reprogramming of myeloid cells by adjuvants and live vaccines deserved to be analyzed in depth. As a consequence, vaccination of elderly people against *influenzae virus* and *Mycobacterium tuberculosis*, using, respectively, anti-*influenzae virus* vaccines with adjuvants and the live vaccine BCG, may enhance their response to Covid-19 (Mantovani and Netea, 2020; Netea et al., 2020b). Therefore, deciphering macrophage heterogeneity induced by different vaccination protocols might help to solve differences in susceptibility to Covid-19 infection.

### Macrophages and Fungal Infections

*Cryptococcus neoformans* (*Cn*) is an opportunistic fungus that infects immuno-suppressed patients. Worldwide, it is still responsible today for more than 1 million life-threatening infections per year (Brown et al., 2012). A recent study identified the presence of two subsets of AMs at an early stage of *Cn* pulmonary infection (Xu-Vanpala et al., 2020). One AM population was CXCL2^+^ and had a pro-inflammatory phenotype whereas the second CXCL2^–^ had an anti-inflammatory phenotype, including expression of IL-10. This anti-inflammatory phenotype was regulated at the epigenetic level. Interestingly, these heterogeneous AM responses were not attributable to fungal inoculum, spatial lung localization, nor ontogeny. This heterogeneity was also confirmed in a model of *Aspergillus fumigatus* infection. Important questions remained, as authors did not explain whether intrinsic or extrinsic cues explained the simultaneous appearance of heterogeneous AM subsets with pro- and anti-inflammatory profiles.

## Macrophage Heterogeneity in Inflammatory Disorders

### Macrophages in Tissue Repair and Fibrosis

The T helper 1 (T_H_1)–T_H_2 paradigm was defined three decades ago (Mosmann et al., 1986), and since then, type 2 immunity was mostly described as a counter-regulatory mechanism dampening type 1 immunity (Wynn and Vannella, 2016). In 2000, Mills and colleagues proposed an elegant parallel between T helper cells and macrophages (Mills et al., 2000). Of note, these two opposite macrophage polarizations were independent of T lymphocytes and were able to influence by themselves opposite immunologic outcomes. Thus, these two distinct populations were termed M1 pro-inflammatory and M2 anti-inflammatory macrophages (Mills et al., 2000). Nowadays, the diverse contributions of type 2 cytokines are more understood. Indeed, in addition to suppressing type 1 response, type 2 immunity and its associated M2 macrophages are well-known contributors of repair and regeneration of injured tissues (Gieseck et al., 2018). Therefore, type 2 response is at the crossroads of two major tasks and its dysregulation can lead to numerous pathological conditions. On the one hand, weak type 2 responses often lead to autoimmune diseases through overstimulation of type 1 responses. On the other hand, chronically activated type 2 responses overactivate wound healing and processes leading to the development of pathological fibrosis (Wynn and Ramalingam, 2012). For many chronic inflammatory diseases, fibrosis is a shared final outcome that can lead to organ failure and death. Fibrotic disorders have been estimated to contribute to 45% of all deaths in the developed world (Wynn, 2004). Numerous fibrotic diseases have been linked to type 2 immunity activation. For instance, chronic helminth infections such as schistosomiasis are associated with fibroproliferative lesions whose mechanism involves type 2 cytokines such as IL-4, IL-5, and IL-13 (Pearce and MacDonald, 2002; Fairfax et al., 2012). As another example, persistence of a chronic injury in the lungs disturbs the wound healing pathways and often leads to fibrosis. Lung fibrosis is of significant medical interest and has been observed in various pulmonary diseases ranging from both acute disorders such as pneumonia, bronchiolitis, ARDS, emphysema, and chronic ones such as idiopathic pulmonary fibrosis (IPF), chronic obstructive pulmonary disease (COPD), asbestosis, asthma, cystic fibrosis, sarcoidosis, and so forth (Gieseck et al., 2018). These diseases affect millions of people globally, and because very few effective treatment options exist, they are one of the leading causes of chronic morbidity and mortality. Type 2 cytokines, as IL-4 and IL-13, are elevated in many of these lung diseases (Grünig et al., 1999; Jakubzick et al., 2003; Keane et al., 2007; Baurakiades et al., 2014; Heitmann et al., 2014; Wills-karp et al., 2016).

Because macrophages and monocytes contribute to the reparation process of injured tissues, from initiation to resolution, several studies suggest a critical role for RTMs in fibrosis pathogenesis. In a model of lung fibrosis, Misharin and colleagues showed that specific depletion of monocyte-derived AMs after their recruitment to the lung ameliorates fibrosis, whereas embryonic AM depletion did not alter fibrosis severity (Misharin et al., 2017). They found that monocyte differentiation to AM occurs progressively during fibrosis and its resolution. Indeed, early monocyte-derived AMs were expressing pro-fibrotic genes, which were then progressively downregulated along their differentiation into mature AMs. Embryonic- and monocyte-derived AMs showed differences in pro-fibrotic gene expression during fibrosis, but ten months later, no more differences were observed. These results revealed remarkable heterogeneity in AM functions according to their origin with important repercussions for the design of innovative myeloid-targeted therapy against fibrosis (Misharin et al., 2017). Our recent work investigated the fibrosis contribution of IMs. First, we demonstrated the existence of two subsets of IMs (LYVE1^hi^MHCII^low^ and LYVE1^low^MHCII^hi^) in distinct sub-tissular pulmonary niches and other tissues. Most importantly, we showed that absence of the LYVE1^hi^MHCII^low^ IMs subpopulation exacerbated immune cell infiltration, tissue inflammation, collagen deposition, and finally fibrotic processes in an experimental model of lung and heart fibrosis. Although these LYVE1^hi^MHCII^low^ IMs are monocyte-derived, they express high levels of genes linked with wound healing and repair and can restrain experimental fibrosis (Chakarov et al., 2019). These two studies highlighted two levels of RTM heterogeneity within the same organ that are important in fibrosis pathogenesis. First, among AMs, ontogeny seems to have an influence as embryonic- and monocyte-derived AMs display different functions. Second, among IMs, the sub-tissular niches seem to have also a significant impact as two IM subpopulations, located in different regions, display different functions. It would be of interest to take into account such heterogeneity for developing innovative therapies in the future.

### Macrophages in Autoimmune Diseases

Rheumatoid arthritis (RA) is a chronic autoimmune disease, in which the small joints are exposed to an inflammatory polyarthritis. RA is a “multicausal” disease that most likely results from a combination of genetic predisposition and various environmental and lifestyle factors. Articular and systemic manifestations in RA can lead to long-term outcomes such as permanent disability. RA is estimated to affect approximately 0.24 to 1 percent of the population (Cross et al., 2014; Hunter et al., 2017). It is defined by a breakdown of tolerance to modified self-protein and chronic synovitis (Lee and Weinblatt, 2001). Current therapies benefit only to a small proportion of patients as sustained clinical remission is only achieved in 20 to 40% of them (Nagy and van Vollenhoven, 2015). However, a minor fraction of patients has long-term drug-free remission for which underlying mechanisms remain ill defined.

Interestingly, the most abundant synovial immune cells of patients in remission are synovial tissue macrophages (STMs). By comparing STMs obtained from patients with active RA, drug-free remission patients, and healthy donors, Stefano and colleagues have identified two STM populations: MerTK^neg^ STMs were enriched in patients with active RA whereas MerTK^pos^ STMs were predominant in drug-free remission patients and healthy donors. MerTK^neg^ STMs had a pro-inflammatory profile, and MerTK^pos^ STMs were negative regulators of inflammation. Interestingly, they identified two subpopulations among MerTK^neg^ STMs: TREM2^pos^ and FOLR2^pos^LYVE1^pos^ STMs. These two subsets reside in different locations and have different and complementary immuno-regulatory roles. Here again, the sub-tissular niches seem to have an impact on RTM heterogeneity. Finally, the authors suggest that therapeutic enhancement of the functions of MerTK^pos^ STMs could facilitate restoration of synovial homeostasis (Alivernini et al., 2020). Another recent study highlighted macrophage heterogeneity in the joint of patients suffering from RA (Culemann et al., 2019). At homeostasis, they identified a subset of TREM2^pos^CX3CR1^pos^ STM that were forming a tight-junction-mediated protective barrier at the synovial lining and physically seclude the joint. These STMs displayed features of epithelial cells and locally proliferated from embryonic-derived interstitial CX3CR1^neg^ macrophages localized within deeper layers of synovial tissue. During RA, this barrier rapidly disintegrated, thus facilitating monocyte infiltration (Culemann et al., 2019). Here again, this study showed very elegantly that ontogeny impacts macrophage function and that RTMs have different roles according to their sub-tissular niche.

### Macrophages in Atherosclerosis

Atherosclerosis is a lipid-driven inflammatory disease where the wall of large arteries is slowly filled by atherosclerotic plaques. Even though it remains an asymptomatic disease in the first half of human life, it remains a major contributor to most cardiovascular diseases affecting elderly individuals. Worldwide, stroke and myocardial infarction are a major social and economic burden and one of the leading causes of death (Lozano et al., 2012). Currently, statins, β-blockers and ACE inhibitors are widely used to control hyperlipidemia and hypertension, respectively. However, efficiency of these treatments is limited.

Macrophages are the most abundant immune cells in the plaque (Cochain et al., 2018; Cole et al., 2018) and have been implicated in all stages of the disease (Hansson and Hermansson, 2011). Recent technical advances in immunology such as cytometry by time of flight (CyTOF) and single-cell RNA sequencing (scRNA-Seq) have enabled a comprehensive mapping of the different macrophages in atherosclerotic plaques (Willemsen and de Winther, 2020). Three main populations of macrophages have been defined: (i) resident-like, (ii) pro-inflammatory, and (iii) foamy TREM2^high^ macrophages (Fernandez et al., 2019). The resident-like macrophages are the only subset in healthy mice but are also present in the adventitia of atherosclerotic aorta. They have an embryonic origin, are self-renewing, express highly FOLR2 and LYVE1, and harbor an anti-inflammatory phenotype (Ensan et al., 2016; Cochain et al., 2018; Kim et al., 2018; Winkels et al., 2018). The pro-inflammatory macrophages are monocyte-derived and are exclusively found in the intima of atherosclerotic aorta where they constitute the largest macrophage subset (Cochain et al., 2018; Kim et al., 2018; Winkels et al., 2018), promoting atherosclerosis lesions. Finally, foamy TREM2^high^ macrophages are monocyte-derived lipid-laden foam cells found exclusively in the intima where they take up atherogenic lipoprotein, resulting in the formation of a lipid-rich core that progresses toward necrotic lesions; they are involved in metabolic regulations and seem to have an immunosuppressive phenotype (Cochain et al., 2018). Here again, ontogeny and sub-tissular niches seems to have an impact on the macrophage polarization and thus on the pathogenesis of the disease.

Another noteworthy fact about atherosclerosis is that it has been epidemiologically associated with infections (Thompson et al., 2013). Indeed, according to numerous human epidemiological studies and animal models, the infectious burden might be linked to later atherosclerotic cardiovascular diseases (ASCVD) and acute infections could cause cardiovascular events (Corrales-Medina et al., 2013; Pothineni et al., 2017). One possible hypothesis to explain such observation is that macrophages and monocytes are trained by the successive infectious challenges throughout life. Such trained immunity provides significant protection against reinfection and improves mortality, even in the absence of an effective adaptive immunity (Kleinnijenhuis et al., 2012; Quintin et al., 2012). However, it also contributes to atherosclerosis progression and to acute disruption of existing atherosclerotic plaques (Christ et al., 2016). Trained monocytes and macrophages display a profound proatherogenic phenotype that is mediated by two intracellular mechanisms which are of metabolic and epigenetic nature. This innate immune memory relies both on central and peripheric modifications, resulting in long-term activation of innate immune cells. On the one hand, several studies showed that bone marrow progenitors such as hematopoietic stem cells (HSC) are indeed subjected to an epigenetic reprogramming upon intravenous BCG vaccination (Kaufmann et al., 2018), intraperitoneal administration of β-glucan, a well-known inducer of trained-immunity (Mitroulis et al., 2018), or intraperitoneal administration of endotoxin (de Laval et al., 2020). On the other hand, Yao and colleagues showed that an innate immune memory induced by adenovirus infection was independent of the contribution of monocytes and bone-marrow progenitors, by taking place directly in resident tissue macrophages (Yao et al., 2018). Across all studies, trained myeloid cells were found to be long-lasting as they were still conferring protection up to months after the initial challenge (Machiels et al., 2017; Kaufmann et al., 2018; Yao et al., 2018; de Laval et al., 2020). Of note, in a very elegant study, Réu and colleagues showed that embryonic RTMs are very long-lasting cells. Indeed, they observed that human microglia were on average 4.2 years old and some of them were found to be more than two decades old (Réu et al., 2017). Such results are of interest because they imply that one single RTM could be challenged several times by different stimuli throughout life, resulting in strong peripheral trained immunity. These results highlight the potential of a better understanding of trained immunity in long-lasting RTM.

To conclude, even if more studies need to be conducted, the epigenetic heterogeneity of macrophages and monocytes might play a significant role in atherosclerosis (Leentjens et al., 2018). Of note, trained immunity can be prevented by pharmacological inhibitors of metabolic pathways, such as glutaminolysis and fatty acid synthesis, and histone methyltransferase blockers (Arts et al., 2016). These could represent innovative strategies to reduce ASCVD risk in patients with acute infections, such as pneumonia. It could also reduce the potential deleterious effects of repeated childhood infections on later ASCVD risk (Leentjens et al., 2018).

## Macrophage Heterogeneity in Metabolic Disorders

### Macrophages in Obesity-Related Insulin Resistance

Nowadays, in high-income countries, a pandemic of obesity threatens the health population by predisposing them to diabetes, non-alcoholic fatty liver disease (NAFLD) and cardiovascular diseases (Heymsfield and Wadden, 2017). For the first time ever, life expectancy is projected to a potential decline (Olshansky et al., 2005), one of the main reasons being the obesity pandemic and all its related deleterious effects (Ludwig, 2016). Overnutrition induces a positive energy balance that leads to fat accumulation in adipose tissue, which triggers immune responses aimed to restore homeostasis.

Adipose tissue macrophages (ATMs) are the largest immune population in adipose tissue and accumulate even more in obesity where they promote a chronic low-grade inflammation (Weisberg et al., 2003; Xu et al., 2003). The long-term consequences of a persistent inflammation are insulin resistance and loss of metabolic flexibility (Reilly and Saltiel, 2017). In obesity, the M1/M2 paradigm presents several limitations and cannot adequately describe ATM functions. Indeed, obesity converts ATMs into a metabolically activated (MMe) macrophage state that is mechanistically distinct from M1-like or M2-like phenotype (Kratz et al., 2014). Coats and colleagues showed that MMe were associated with both production of inflammatory cytokine (a harmful function) and clearance of dead adipocytes by lysosomal exocytosis in crown-like-structure around dying cells (a beneficial function) (Coats et al., 2017). Traditionally, these opposite functions were attributed to distinct ATM subpopulations: the detrimental one being associated with M1-like ATMs while the beneficial ones being ascribed to M2-like ATMs. However, Coats and colleagues provide evidence that these two functions were the properties of a single MMe macrophage subset that evolves upon diet-induced obesity. These findings were confirmed in a following study using a single-cell sequencing approach. The authors were able to identify a population of CD9^+^ ATMs that localized to crown-like structure, were enriched in lipids, and upregulated both inflammatory pathways and lysosomal metabolism (Hill et al., 2018). Finally, a recent study identified one population of CD9^+^TREM2^+^ lipid-associated macrophages (LAMs) that arise in obesity conditions from recruited monocytes and formed crown-like structure. These LAMs were able to prevent adipocyte hypertrophy, hypercholesterolemia, inflammation, body-fat accumulation, and glucose intolerance (Jaitin et al., 2019). Together, these results highlight the limitations of the M1/M2 paradigm and showed that one same long-lived macrophage can harbor opposite functions across time, according to the duration of the challenge.

### Macrophages in Non-alcoholic Fatty Liver Disease (NALFD) and Its Inflammatory Form, Non-alcoholic Steatohepatitis (NASH)

NAFLD is defined by an excessive fat accumulation in the liver and is associated with obesity and metabolic syndrome. In the United States, 25–30% of the population develops NAFLD, which then may progresses into a more serious form of NAFLD, termed non-alcoholic steatohepatitis (NASH), characterized by chronic liver injury, fibrosis, and inflammation (Cohen et al., 2011; Diehl and Day, 2017; Samuel and Shulman, 2018). NASH can subsequently cause end-stage liver pathologies, as cirrhosis and hepatocellular carcinoma (HCC). Finally, it is a common indication for liver transplantation (Pais et al., 2016; Diehl and Day, 2017).

A recent study discovered specific NASH-associated macrophages (NAMs) that were present both in mice and in humans (Xiong et al., 2019). These NAMs represent about 60% of macrophages from NASH livers; they expressed high amount of TREM2 and CD9 and appear to have a protecting role during NASH pathogenesis. Another study identified in cirrhotic patients a subpopulation of scar-associated macrophages (SAMacs) that differentiates from circulating monocytes, expresses a high amount of TREM2 and CD9, and had a pro-fibrotic phenotype. These SAMacs were also expanded in a cohort of patients suffering from NASH (Ramachandran et al., 2019). Thus, even if macrophages are heterogeneous from one organ to another, it appears that they also share similar properties as illustrated by TREM2 signaling in adipose tissue and the liver. Interestingly, two very recent studies confirmed these findings in mice (Remmerie et al., 2020; Seidman et al., 2020). Indeed, in a model of metabolic-associated fatty liver disease (MAFLD), Remmerie et al. showed that KCs were progressively eliminated along the course of the disease and slowly replaced by monocyte-derived cells. A subset of them was termed “hepatic LAMs” as they had a transcriptome similar to adipose tissue LAMs and fibrotic liver SAMacs (Remmerie et al., 2020). In addition, Seidmann et al. showed that within the diseased liver, different microenvironments are responsible for distinct differentiations among resident and infiltrating immune cells by remodeling the chromatin status of recruited monocytes but also by modifying the activities of preexisting enhancers of the resident KC population (Seidman et al., 2020).

## Macrophage Heterogeneity in Age-Related Disorders

### Macrophage, Immuno-Senescence, and Inflammaging

Thanks to modern medicine and public health measures, human life expectancy is far better today than it was a century ago. However, a prolonged lifespan goes along with a rise in non-communicable diseases such as cancer and cardiovascular, autoimmune, and neurodegenerative diseases and a higher susceptibility to infections. As a consequence, the aging research community has seen the emergence of geroscience, a research field that aims to extend human longevity (Kennedy et al., 2014). The immune system is impacted by virtually all hallmarks of aging (López-Otín et al., 2013) by undergoing with age a profound remodeling termed immuno-senescence that impacts both arms of our immunity (Grubeck-Loebenstein et al., 2009).

Immuno-senescence is generally associated with a loss of immune functions and defective immune system. All immune cells are affected, ranging from a dysfunction of adaptive T and B cells to functional changes of innate immune cells subsets, such as monocytes and macrophages (Grubeck-Loebenstein et al., 2009). For instance, although the absolute number of monocytes is constant upon aging, the ratio of monocyte subsets is altered: classical monocytes (CD14^+^CD16^–^) are reduced, while intermediate (CD14^+^CD16^+^) and non-classical monocytes (CD14^low^CD16^+^) are increased (Seidler et al., 2010; Hearps et al., 2012). Of note, non-classical monocytes have a lower expression of HLA-DR suggesting a decline of antigen presentation function. Likewise, aged macrophages exhibit a lower level of MHC-II expression (Herrero et al., 2002), have a disabled clearance of dead cell capacity (Aprahamian et al., 2008) and a reduced chemotaxis (Solana et al., 2012). However, some innate immunity features seem to be conserved or even increased during immuno-senescence (Franceschi et al., 2000a). Of note, inflammation is not reduced upon aging, and a low-grade, chronic, sterile inflammation, called “inflammaging,” seems to be a conserved phenomenon in elderly patients (Franceschi et al., 2000b; Oishi and Manabe, 2016). Indeed, serum levels of IL-6 and CRP increase with age, and their levels are associated with a decline of physical and cognitive performance and predict mortality in the elderly (Puzianowska-Kuźnicka et al., 2016). Human monocytes from the elderly have been shown to express more TNFα (Hearps et al., 2012) and to produce more TLR5-induced IL-8 and IFN-γ-mediated IL-15 (Qian et al., 2012; Lee et al., 2014). In addition, it has been proposed that danger-associated molecular patterns (DAMPs) accumulation, a central driver of inflammation, could be linked with the age-related decline of phagocytic and autophagy activities in macrophages (Oishi and Manabe, 2016; Bulut et al., 2020). Finally, upon aging, senescent cells secrete several inflammatory chemokines and cytokines, a phenomenon termed as the senescence-associated secretory phenotype (SASP). Hall and colleagues have shown that senescent cells can reprogram macrophages, hence termed senescent-associated macrophages (SAMs) (Hall et al., 2016, 2017). SAMs were p16^+^ and β-gal^+^, two reliable markers of senescence and displayed both M2-like phenotype and pro-inflammatory profile.

Another contributor of macrophage heterogeneity is clonal hematopoiesis, a process by which genetically distinct subpopulations can be generated from HSC that have underwent DNA point mutations (Beerman et al., 2010). Indeed, it has been widely observed that HSC-derived monocytes gave rise to RTMs upon aging and related accumulated challenges. In addition to addition to leading to the onset of many diseases including blood cancers (Genovese et al., 2014; Jaiswal et al., 2014), this phenomenon could also generate mutated RTM subpopulations involved in specific diseases, cancer notably but also neurodegenerative diseases.

Thus, macrophage heterogeneity will never stop from increasing as we get older. From an innate adaptative point of view, the whole life is a series of successive exposures to various antigens, each of them having an impact on innate immune cells. As a consequence, type, intensity, and temporal sequences of antigen exposure are directly linked to the trained immunity. Recently, the combination of these elements has been called immunological biography or “immuno-biography” (Franceschi et al., 2017). This immuno-biography is considered to be unique for each individual, each one of us having a unique set of heterogeneous trained macrophages. Although still theoretical, medical specialties such as gerontology and geriatrics should pay particular attention in the future, to the immunological anamnesis of each individual to reconstruct their own immuno-biography and predict their subsequent immune responses. However, this will take time before reaching hospital practices as limitless data are impactful and nearly every challenge should be collected: type of delivery (natural *vs.* caesarian), of diet and early nutrition (breast or industrial milk), of infectious diseases and vaccinations, socioeconomic context, ethnicity, psychological status, use of antibiotics, composition of microbiota, and suchlike.

### Macrophages in Neurological Diseases

The burden of neurological disorders is increasing as populations are growing and aging. In 2016, disorders of neurological origin were the leading cause of disability-adjusted life years (DALYs) and the second leading cause of deaths. The four largest contributors of neurological DALYs are strokes, migraines, the spectrum of Alzheimer’s related dementia and meningitis (Feigin et al., 2019). In the CNS, neuronal and non-neuronal cells are working together. Among non-neuronal cells, “glia” are composed of astrocytes, oligodendrocytes and microglia (Castellani and Schwartz, 2020). As current progresses acknowledge the role of innate immunity and neuroinflammation in driving neurodegenerative disorders, brain-resident macrophages, i.e., microglia, have taken central stage (Heneka et al., 2018; Lenz and Nelson, 2018). Microglia originate from embryonic-yolk-sac precursors and are self-renewal at steady state. Their apparent heterogeneity has raised several questions regarding their distinct roles in health and diseases (Ginhoux et al., 2010; Ennerfelt and Lukens, 2020).

In a model of neonatal stroke, a recent study reports the contribution of monocytes to microglia during inflammation by using a new mouse fate-mapping model that labels monocyte derivatives (Chen et al., 2020). After neonatal brain ischemia, CCR2^+^ monocytes localized at the ischemic border but were also found in distant peri-infarct sites. At first, these recruited monocytes had an ameboid cell shape and a pro-inflammatory phenotype, but then changed to a more ramified morphology that resemble microglia at day 30, along with the upregulation of microglial gene signatures and M2-like markers. These results suggest a dual function of monocytes after neonatal strokes – i.e., the exacerbation of acute brain damages followed by resolution of inflammation. In addition, they highlighted that infiltrating monocytes undergo *in situ* reprogramming in the brain in order to contribute to the pool of microglia.

Neurodegeneration is defined by an age-related progressive loss of neurons in the central nervous system (CNS), leading to alterations of cognitive performance and dementia (Ramanan and Saykin, 2013). Alzheimer disease (AD) is a neurodegenerative disease with no efficient treatment, characterized by prominent neuroinflammation, extracellular accumulation of amyloid-β (Aβ), and deposition of neurofibrillary tangles in neurons (Castellani and Schwartz, 2020). Here again, microglia have key roles in its pathogenesis. Interestingly, genome-wide association studies (GWAS) in patients with AD have linked mutations in microglial pattern recognition receptors (PRR), including TREM2, with diseased risk (Mhatre et al., 2015). Recently, a new subset of microglia termed “disease-associated microglia” (DAMs) has been identified both in mice and in humans (Keren-Shaul et al., 2017). DAMs expressed genes, which were found associated with AD in human GWAS (Lambert et al., 2013; Keren-Shaul et al., 2017). Genes involved in lipid and metabolic pathways as well as lysosomal and phagocytic capacities are upregulated, including known risk factors of AD, such as APOE and TREM2 (Lambert et al., 2013; Krasemann et al., 2017). DAMs were first detected in the diseased CNS regions, but not in healthy ones. In murine models of AD, DAMs colocalize with Aβ plaques (Keren-Shaul et al., 2017; Mrdjen et al., 2018). Markers of DAM signature were also observed in human AD *postmortem* brains (Friedman et al., 2018). DAMs were shown to be heterogeneous across time as their differentiation appears to be a sequential two-step process: first, microglia shift toward a DAM stage 1 which then moves to a DAM stage 2 (Keren-Shaul et al., 2017; Friedman et al., 2018). This microglial shift, from a homeostatic phenotype to a DAM signature, is believed to rely on the sensing of neurodegeneration-associated molecular patterns (NAMPs). NAMPs, such as Aβ, are danger signals commonly present in various brain conditions, and they are recognized by microglial PRR, such as TREM2 (Deczkowska et al., 2018). TREM2 is a very well-characterized PRR involved in the pathogenesis of AD. Engagement of TREM2 stimulates myeloid cell survival, as well as cytoskeletal reorganization and pro-inflammatory cytokine production (Ulland and Colonna, 2018). Mutations of TREM2 have been reported in patients suffering from late-onset AD (Guerreiro et al., 2013; Jonsson et al., 2013). Several studies have found a beneficial role of TREM2 in Aβ sensing and clearance in various experimental models (Wang et al., 2015, 2016; Ulland et al., 2017; Zhao et al., 2018; Parhizkar et al., 2019). The DAM response is believed to be a protective mechanism aiming at containing neuronal damages, even if several studies are still showing conflicting results, likely due to the heterogeneity of DAMs, as their origin is still yet unclear. However, the discovery of DAMs creates opportunities to develop therapies targeting universal mechanisms of fighting against neuronal death shared by several neurodegenerative conditions (Deczkowska et al., 2018).

## Macrophage Heterogeneity in Neoplastic Disorders

Among non-communicable diseases, cancer, and all its associated spectrum of diseases, ranks as the leading cause of death (World Health Organization, 2018). Cancer incidence and mortality are still growing worldwide. In 2018, there were an estimated 18.1 million new cases of cancers and 9.6 million deaths from cancers (Bray et al., 2018; Ferlay et al., 2019). Globally, 1 in 6 deaths is due to cancer. In both sexes, lung cancer is the leading cause of cancer death, followed by female breast cancer for incidence and colorectal, stomach, and liver cancers in terms of mortality. In addition, some cancers, such as pancreatic and brain cancers, are less frequent and thus account for a small absolute number of deaths but have a very low five-year survival rate as compared to other cancers like breast cancer.

The emergence of immuno-therapies targeting checkpoint inhibitors during the last decades constituted a major breakthrough in oncology treatment by significantly improving patient prognosis (Couzin-Frankel, 2013). Several inhibitors have reached a market authorization for various cancers, and numerous clinical trials are ongoing worldwide. However, such therapies are expensive, have immune-related adverse events (irAEs) and despite many clinical objective responses, not all patients are responders. Therefore, both clinical and fundamental studies are still urgently needed. Here again, macrophages, called in this context tumor-associated macrophages (TAMs), are the predominant immune cells in the tumor microenvironment (TME) and play a fundamental role in tumor biology.

### Macrophages and Lung Cancer

In 2018, 2.1 millions of patients developed a new lung cancer. There were 1.8 million deaths, representing almost 1 in 5 deaths by cancer. The 5-year survival of lung cancer is disastrous, as it is comprised between 15 and 20%. In a mouse model of lung cancer, TAMs have been shown to have a dual origin: they derive both from resident interstitial macrophages (IMs) present before tumorigenesis and from adult monocytes recruited after tumors start to expand (Loyher et al., 2018). However, it has been recently shown that resident IMs also derive from adult monocytes, whereas AMs are from embryonic origin (Chakarov et al., 2019). As it was shown that AMs were not significant contributors of TAM population in lung cancer (Loyher et al., 2018), we can hypothesize that TAMs actually originate exclusively from adult monocytes, with a varying time of residency within the tissue. In patients suffering from non-small cell lung carcinoma (NSCLC), another recent study highlighted a spatial heterogeneity between TAMs located in the tumor core and TAMs located at invasive margin (Zheng et al., 2020). Indeed, pro-tumoral TAMs were marked especially at the tumor-invasive margin. Moreover, pro-tumoral TAMs were in closer contact to tumor cells as compared to the antitumoral ones. Finally, at the invasive margin, higher proximity of tumor cells to pro-tumoral TAMs and lower proximity to antitumoral TAMs were associated with poor survival (Zheng et al., 2020). Finally, another recent study of human and mouse NSCLC identified a new population of mature dendritic cells enriched in immuno-regulatory molecules (mregDCs) that limit antitumor immunity (Maier et al., 2020). As we can expect to find similar or related programs in TAMs, research in tumoral macrophages needs to be pursued.

### Macrophages and Breast Cancer

Worldwide in 2018, 2.1 million newly female breast cancers were diagnosed, accounting for almost 1 in 4 cancer cases among women. A meta-analysis showed that high density of TAMs correlates with poor survival rates and suggested to use TAM density as a prognostic factor (Zhao et al., 2017). Consistent with a pro-tumoral role of TAMs, genetic ablation of *Csf-1* in a murine model of breast cancer (resulting in ablation of macrophages) resulted in delayed tumor development and reduced pulmonary metastasis (Lin et al., 2001). Regarding ontogeny, in a mouse model of mammary tumor (MMTV-PyMT), Franklin et al. showed that TAMs were strictly derived from recruited inflammatory monocytes (Franklin et al., 2014). Interestingly, a study showed in a model of mouse breast cancer, that in response to CCL2 secretion from tumor cells, stromal macrophages were recruited, became intra-epithelial macrophages and induced Wnt-1 production to dismantle E-cadherin junctions, thus promoting early cancer cells dissemination (Linde et al., 2018). In parallel, a recent study (Dawson et al., 2020) identified a new population of ductal macrophages in ductal epithelial structures that were different from the resident stromal macrophages. These ductal macrophages were monocyte-derived and constantly monitored the epithelium throughout breast oncogenesis. Of note, by comparing healthy human breast tissue *vs.* human tissue with lesions of ductal carcinoma *in situ* (DCIS), macrophages were found inside aberrant ductal epithelial structures in between cancer cells that showed reduced E-cadherin levels. Finally, high-grade lesions contained more intra-epithelial macrophages as compared to healthy and low-grade DCIS (Linde et al., 2018).

Besides, another very recent study showed that modulation of TREM2 had a remarkable impact on TAM landscape (Molgora et al., 2020). As in some diseases discussed ahead, TREM2 has already been reported in tumors (Lavin et al., 2017; Song et al., 2019). Indeed, authors showed that *Trem2*^–/–^ mice were more resistant to tumor growth than WT mice in a mammary tumor mouse model. *Trem2* deficiency showed alterations in macrophage populations and an increase of tumor-infiltrating CD8^+^ T cells expressing PD-1. Of note, authors showed that anti-PD-1 therapy was more effective in *Trem2*-deficient mice than in WT mice. Furthermore, anti-TREM2 mAb dampened tumor growth and highly enhanced the efficiency of anti-PD-1 immunotherapy. *Trem2* deficiency and anti-TREM2 mAb were responsible for changes in the tumor-infiltrating macrophages: CX3CR1^+^ and CD206^+^ macrophage subsets declined, while other subsets were induced. Lastly, authors found that TREM2 was a marker of TAM in more than 200 human tumors and that its expression was inversely correlated with greater relapse-free survival and overall survival (OS) and in triple-negative breast cancer. To sum up, TAM remodeling by specifically targeting TREM2 could be a promising avenue for complementing checkpoint immuno-therapy (Molgora et al., 2020). A concomitant study also identified a TREM2^+^ regulatory monocytes in a model of mouse fibrosarcoma (Katzenelenbogen et al., 2020). By coupling scRNA-Seq and intracellular protein activity, authors showed that this population was associated with more dysfunctional CD8^+^ T cells and tumor growth.

### Macrophages and Pancreatic Cancer

Although not the most frequent, pancreatic cancer is associated with one of the worst prognoses. In mice, in a model of pancreatic ductal adenocarcinoma, TAM has been shown to originate from both embryonic-RTMs and recruited inflammatory monocytes (Zhu et al., 2017). Embryonic TAMs exhibited a pro-fibrotic profile with increased expression of genes involved in extracellular matrix deposing and remodeling, which is a hallmark of pancreatic ductal adenocarcinoma. In contrast, monocyte-derived TAMs were more efficient antigen-presenting cells (Zhu et al., 2017). Here again, such results argue for a role of macrophage ontogeny in tumor pathogenesis and these findings should be taken into account for future TAM-targeted therapies.

### Macrophages and Liver Cancer

Liver dysfunctions from NAFLD to NASH, cirrhosis, and hepatocellular carcinoma (HCC) account for 2 million deaths per year (Williams et al., 2014). HCC was the second leading cause of years of life lost (YLLs) from cancer worldwide between 2005 and 2015 (Yang et al., 2019). According to the staging, various treatments are recommended, ranging from surgical resection to systemic chemotherapy (Marrero et al., 2018). We have recently observed that a subpopulation of FOLR2^+^ TAMs underwent an onco-fetal reprogramming, meaning that these adult cells can acquire a transcriptomic profile similar to fetal macrophages in the specific context of liver cancer. Considering that fetal macrophages are strongly tissue-supportive and take part in the organism development, the reprogramming could explain why TAMs are pro-tumoral. Of note, this phenomenon, mediated by tumoral endothelial cells (ECs) (Sharma et al., 2020), is only partial and does not concerned other TAM subpopulations, such as SPP1^+^ and MT1G^+^ TAMs. Although therapeutic strategies are yet to be designed to modulate this onco-fetal reprogramming, these results unambiguously demonstrate that TAM-oriented immunotherapies need to be very precisely designed in order to be specific and efficient with limited side effects.

### Macrophages and Brain Cancer

Brain cancer accounted for only 2.5% of all deaths by cancer in 2018 worldwide. However, as in the pancreas, the five-year mortality rate is disastrous. Studies have identified two TAM subsets in human glioma: one was from embryonic origin and the other one originated from adult bone marrow monocytes (Bowman et al., 2016; Müller et al., 2017). Muller et al. showed that monocyte-derived TAMs, but not embryonic TAMs, were correlated with shorter OS in low-grade glioma. Interestingly, Bowman et al. identified *Itga4* (*Cd49d*) as an effective marker to distinguish embryonic TAMs from monocyte-derived TAMs TAMs both in mice and in humans. They also showed that while embryonic- and monocyte-derived TAMs shared features of tumor education, they exhibited distinct activation states: embryonic TAMs were enriched in pro-inflammatory genes as well as factors involved in extracellular matrix (ECM) remodeling, while monocyte-derived TAMs exhibited an immuno-suppressive signature. Their data suggest that these different faculties resulted from inherent transcriptional networks poised before the onset of tumorigenesis. Therefore, as these chromatin landscapes were established earlier of tumor initiation, it suggests that ontogenically unrelated cells can be engaged in distinct macrophage activation states (Bowman et al., 2016). In addition, Muller et al., showed that embryonic- and monocyte-derived TAMs were enriched in distinct tumor-anatomical structures and that both of them had a gene signature of both M1- and M2-like (Müller et al., 2017).

### Macrophages and Metastasis

Macrophages are key players in the formation of pre-metastatic niches (Lopez-Yrigoyen et al., 2020). Indeed, in the primary tumor, they help tumor cells to escape from immune recognition and they prepare distant “pre-metastatic” sites for tumor cells to colonize (Paget, 1889). These “pre-metastatic” niches are shaped by systemic influences of primary tumor through recruitment of monocytes that in turn attract tumor cells by chemokines. Macrophages also remodel the ECM to promote angiogenesis, epithelial-to-mesenchymal transition, and extravasation. Therefore, they enhance both tumor cell tropism and their ability to seed and survives (Psaila and Lyden, 2009). Once tumor cells arrive at these “pre-metastatic” sites, a distinct subset of macrophages termed metastasis-associated-macrophages (MAMs) promotes tumor cell extravasation and growth (Qian et al., 2011). Therefore, MAMs derive exclusively from monocytes, are pro-tumoral (Kitamura et al., 2018), and have been shown to limit the efficacy of classical cancer therapies such as chemotherapy, radiotherapy, and biological therapies (Lopez-Yrigoyen et al., 2020).

### Macrophages and the 3 E’s Theory

In the early twentieth century, Paul Ehrlich conceived the idea that the immune system could suppress an “*overwhelming frequency*” of carcinoma (Ehrlich, 1909). The revisiting of the Ehrlich proposal had to await the maturation of immunology, and the concept of “*cancer immuno-surveillance”* was proposed in 1957 by Burnett and Thomas. It was defined as follows: *“In large, long-lived animals, like most of the warm-blooded vertebrates, inheritable genetic changes must be common in somatic cells and a proportion of these changes will represent a step toward malignancy. It is an evolutionary necessity that there should be some mechanism for eliminating or inactivating such potentially dangerous mutant cells and it is postulated that this mechanism is of immunological character*.” Subsequently, the immuno-surveillance concept was hardly challenged because numerous *in vivo* experiments failed to prove it. Therefore, this theory was rapidly forgotten and relegated to the historical dustbin. A major review published in 2000, which listed the six critical hurdles that a new tumor must circumvent to grow and survive, did not even mention the basal immune response against tumors (Hanahan and Weinberg, 2000). However, because of growing progress in immunology and comprehension of some limitations of mouse model, the new millenium has witnessed the revival of this old debated idea. A second major review published 11 years later by the same authors added 4 supplemental hallmarks. Among them were (i) the avoidance of immune destruction and (ii) the tumor-promoting inflammation by innate immune cells (Hanahan and Weinberg, 2011). Since then, the immuno-surveillance hypothesis has shifted to the “cancer immuno-editing” concept that proceeds through three phases termed “the three Es” for elimination, equilibrium, and escape (Dunn et al., 2002). Although the role of NK and T cells has been very well defined in these processes, TAM implication should also be taken into account.

**FIGURE 2:**
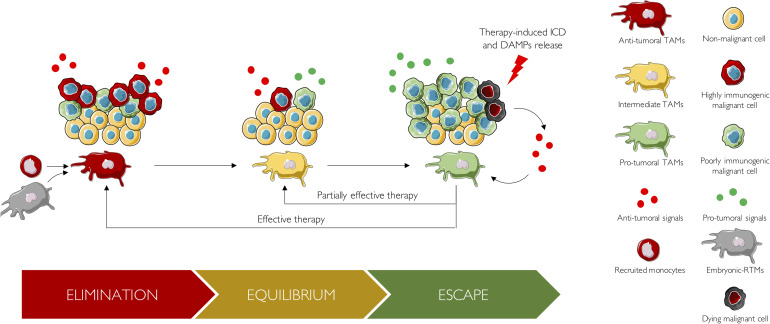
The 3 E’s of cancer immuno-editing from a TAM perspective. During the elimination phase, TAMs are antitumoral and collaborate with adaptive immune cells to eliminate highly immunogenic tumor cells. These TAMs arise either from recruited monocytes or from embryonic RTMs. Thereafter, tumor cells able to survive the elimination step, proceed to the equilibrium phase, a state of tumor quiescence where net growth is limited and cellular immunogenicity edited. During this phase, tumor cells slowly influence the TME to provide support for their growth: TAMs are slowly educated to enhance tumor progression and switch from an antitumoral state to a pro-tumoral one. Pro-tumoral cues responsible for TAM reprogramming, as well as the role of ontogeny, should be studied in further details. Finally, edited tumors enter the escape phase. There, tumor growth becomes unrestrained, tumors are clinically detected, and patients are receiving their first line of treatment. In case of treatment failure, no objective response is observed, and tumor eventually stays in the escape phase. Alternatively, a partially effective therapy, might drive tumor into an on-treatment equilibrium phase, which would be characterized by a partial response. Finally, effective therapies can drive tumors back to the elimination phase, which will be reflected by a complete response. Upon treatment, tumor cells undergo immunogenic cell death (ICD) and released DAMPs. These DAMPs boost anticancer immune response by reprogramming antitumoral TAMs into pro-tumoral TAMs.

Indeed, revisiting the 3 E’s theory of tumor immuno-editing through the lens of TAM biology is an interesting way to highlight their incredible heterogeneity through time (Figure 2). During the earliest stage of tumor onset, i.e., the elimination phase, TAMs are antitumoral and collaborate with adaptive immune cells to recognize and eliminate tumor cells (Dunn et al., 2004). Here, TAM ontogeny remains only partially characterized and should be further investigated to understand its precise contribution to this antitumoral phase. Thereafter, tumor cells able to survive the elimination step can proceed to the equilibrium phase, a state of tumor quiescence where net growth is limited, and cellular immunogenicity edited. During this phase, tumor cells slowly influence the TME to provide support for their growth. There, the majority of TAMs are slowly educated to enhance tumor progression and switch from an antitumoral state to a pro-tumoral one. Of note, ontogeny may have a role in this phenotype shift. Indeed, embryonic TAMs and monocyte-derived TAMs may exhibit different epigenetic profiles that could influence their subsequent education by tumor cells. Thus, understanding the role of ontogenic dimension and the tumoral cues that underlies this antitumoral to pro-tumoral transformation would help blocking or delaying TAM education, which is of course of major interest for preventive medicine. Finally, edited tumors enter the escape phase, where their growth becomes unrestrained and become clinically detected. Patients are generally receiving their first line of treatment during this escape phase. Studies of patients treated with immuno-therapies indicate that the immuno-editing process can reoccur in response to treatment (O’Donnell et al., 2019). Indeed, in case of treatment failure, no objective response is observed, and tumor eventually stays in the escape phase. Alternatively, a more efficient but still inadequate treatment might drive tumor into an on-treatment equilibrium phase, which would be characterized by a partial response. Finally, effective therapies can drive tumors back to the elimination phase, which will be reflected by a complete response.

Interestingly, it is now well known that antineoplastic agents such as anthracyclines, oxaliplatin, and crizotinib stimulate the liberation of DAMPs, from dying cancer cells via the induction of immunogenic cell death (ICD) (Galluzzi et al., 2017). Once released, DAMPs operate as immunological adjuvants and boost anticancer immune responses by converting “cold” into “hot” tumors, characterized by accumulation of pro-inflammatory cytokines and T cell infiltration, resulting in a better response rate to immune-checkpoint blockers (Galluzzi et al., 2017). Numerous DAMPs released by ICD have been already described, such as HMGB1 and ATP (Galluzzi et al., 2020). They have been shown to induce a shift from pro-tumoral to antitumoral TAM (Li et al., 2017). In addition, a very recent study showed that radiation induces the release of microparticles from tumor cells, which induces as well the reprogramming of TAM polarization from a pro-tumoral to an antitumoral phenotype (Wan et al., 2020). To sum up, TAMs are a highly plastic and very heterogeneous population able to change across time as the tumor evolves and upon challenges such as therapies.

## Concluding Remarks

Since their first description by Ilya Ilitch Metchnikov in the late 19th century (Metchnikov, 1883), macrophages have become the focus of many studies. A lot of knowledge has been accumulated over the years shaping our actual understanding of these immune cells in both healthy and diseased tissues. The current paradigm, written in textbooks and taught to students, is that macrophages are phagocytes in charge of the tissue immune surveillance that they exert by ingesting every foreign particle they can catch, making them the prototypal innate immune cells. For this reason, macrophages for which phagocytosis represents the main function are often considered as archaic cells, only assigned as pro-(M1) or anti-(M2) inflammatory and are too often overlooked in the design of innovative therapeutic strategies. Our aim herein was to rehabilitate them as central players of tissue dysbiosis observed in almost all types of human diseases. Indeed, macrophages represent often the most abundant immune cell population in diseased tissues. Furthermore, their inherent plasticity allows them to display a multitude of phenotypes, either supporting or restricting disease development. So, clearly a global strategy neutralizing or depleting macrophage population as a whole is no more conceivable based on the current knowledge. It is now time to target more specifically macrophage populations that support pathogenesis. This could only be done by clarifying the major programs shaping macrophage biology in a time- and spatial-dependent manner. For this, global integration of existing data is needed and is actually in progress in many laboratories and will definitely improve our fundamental knowledge and upcoming therapeutic strategies.

## Author Contributions

GG, CB, and FG discussed and established the plan of the manuscript and approved the final version for publication. GG and CB wrote the draft. FG provided a substantial intellectual contribution and edited the manuscript. All authors contributed to the article and approved the submitted version.

## Conflict of Interest

The authors declare that the research was conducted in the absence of any commercial or financial relationships that could be construed as a potential conflict of interest.
